# Supplementary material for: Thousands of reptile species threatened by under-regulated global trade
Source: Nat Commun. 2020 Sep 29;11:4738. doi: 10.1038/s41467-020-18523-4 (PMC7525537; doi:10.1038/s41467-020-18523-4)
Supplement: Supplementary file 1 — Supplementary Information [file 41467_2020_18523_MOESM1_ESM.pdf]

Supplementary Materials

Includes:

Supplementary Discussion 1
Supplementary Figures 1-10
Captions for Data 1-7, and Supplementary Software 1

Supplementary Discussion 1

Caveats and technical limitations

While already alarming, the true extent of the under-documented live reptile trade is likely more extensive than the results we present. Our methods miss two venues of online trade —the dark web and social media. Previous investigations into the wildlife trade on the dark web have concluded that trade is currently limited in scope (1, *2*), suggesting that the lack of enforcement on the open web does not necessitate more drastic measures to hide exchanges (3). In contrast, social media has been implicated as an enabler of wildlife trade (4). Efforts to leverage machine learning to detect and document illegal trade have shown some success (4-7). However, these efforts may be frustrated by code-words (*8*) or limited access (9).

Temporally, our investigations were limited by the snapshots available on the Internet Archive. We are unable to assess any biases associated with when or how these snapshots were generated. Further, we were limited as other species-rich sites did not have coverage over a long enough period to examine trends. These limitations do not undermine our findings, rather they support the assertion that the reptile trade covers more species than we were able to detect, and when the new species detected in each language, site, and year are considered it must be concluded that an even larger proportion of species are in trade.

Outside of sampling, it is worth considering sources of potential false positive and negative species detections. False positives, the detection of a species not currently traded, are likely a smaller source of error compared to false negatives. False positives may result from naming issues, either by incorrect naming of species by traders, or a common name referring to a number of different species. Listing of species only to the genus level or using older naming conventions could confuse the exact species traded. Identity confusion could be especially apparent in cryptic species, for example Ratsoavina et al. (10) suggest that *Uroplatus finaritra* has likely existed in the trade described as a giant variety of *U. phantasticus*. Unpicking the exact identity of a traded species could be nearly impossible without locality information.

Second is the overlap of common names, *i.e.*, one common name that is used to describe multiple species. There are several examples of non-unique or encapsulated common names in the dataset –for example, 'Madagascar giant day gecko' is a common name for *Phelsuma grandis* but encapsulates 'Giant day gecko' that is connected to *Phelsuma gigas*– but the high number of species confirmed by scientific name suggest that the commonality of common names is not overly inflating species numbers. However, scientific names are not immune to being widespread as large species complexes can confuse the identification of species. For example we detected the newly described *Cyrtodactylus bokorensis* only via a name (*Cyrtodactylus intermedius*) assigned prior to the species complex split that produced *C. bokorensis*. Cases like this lead would inflate the species detected in the trade but are likely countered by synonymisations. For example, a raw count of unique species names from the all sources suggests 4444 species, but when matched to accepted Reptile Database this number falls to 3943 as a result of synonymisations and listings only to a genus level.

False negatives –the failure to detect a species traded– are likely a larger source of error in our methods. Due to computational limitations we were restricted to fixed string matching, this method is sensitive to small differences in the display of diacritical marks. Any name with a different way of displaying diacritical marks to our set of keywords would not be detected. We would also fail to detect species when names are split by other details or incomplete, for example an albino leopard gecko being listed as ‘leopard albino’. For Japanese sites especially –for which we had no common names– our common name list is not comprehensive, missing species described only by local/common names.

Supplementary Figures

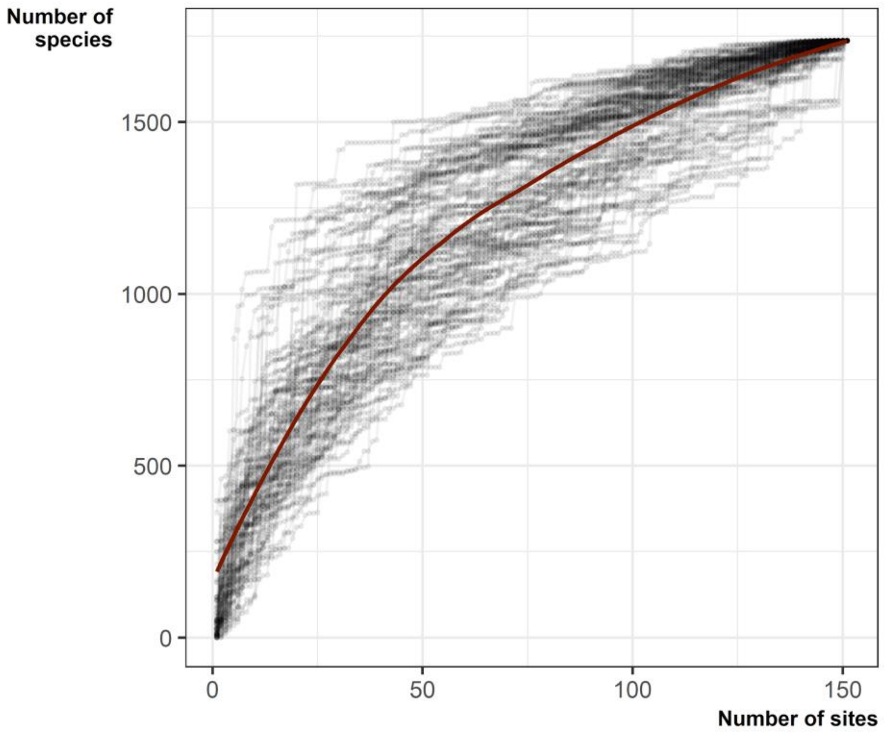

Supplementary Figure 1. Resampled accumulation curve showing the relationship between the number of species detected in the 2019 snapshot and the number of websites searched.

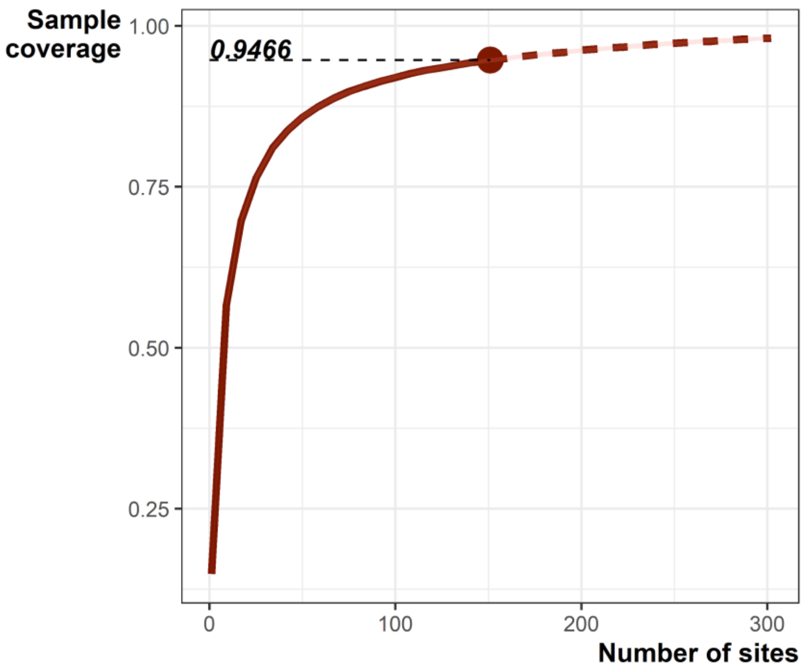

Supplementary Figure 2. Sample completeness curve for the 2019 snapshot dataset covering species detected on trade websites.

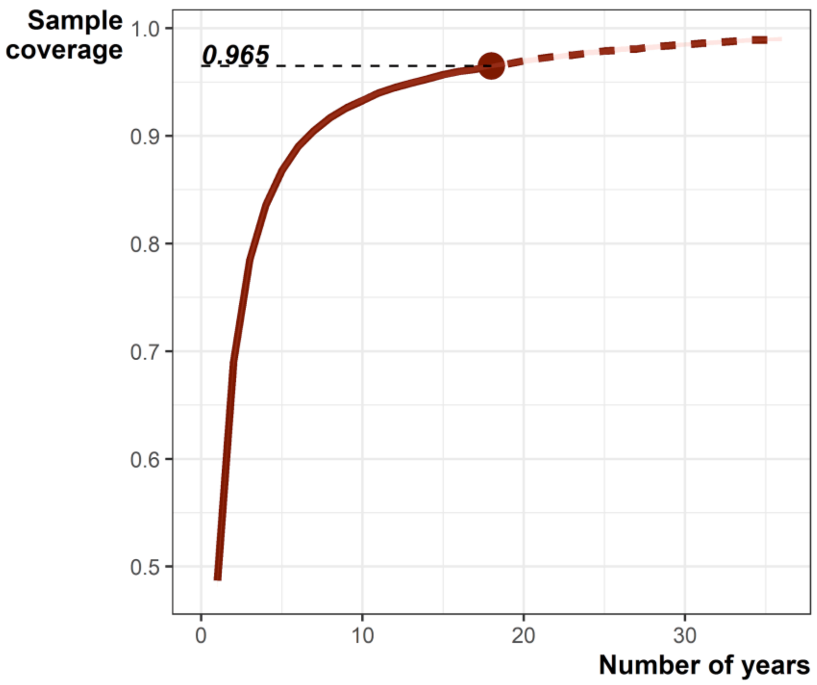

Supplementary Figure 3. Sample completeness curve for the 2002-2019 temporal dataset covering species detected on trade websites.

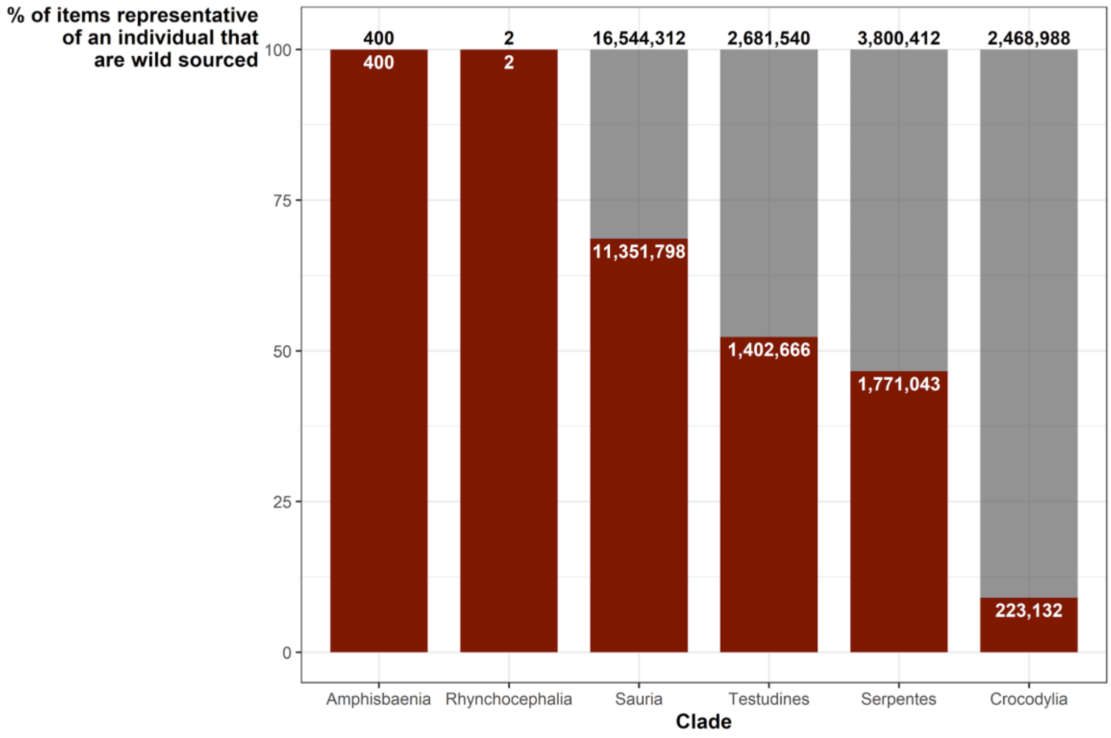

Supplementary Figure 4. The percentage of individuals sourced from the wild in LEMIS data 2000-2014. White numbers show the number of traded items from the wild, the black numbers describe the total number of traded individuals.

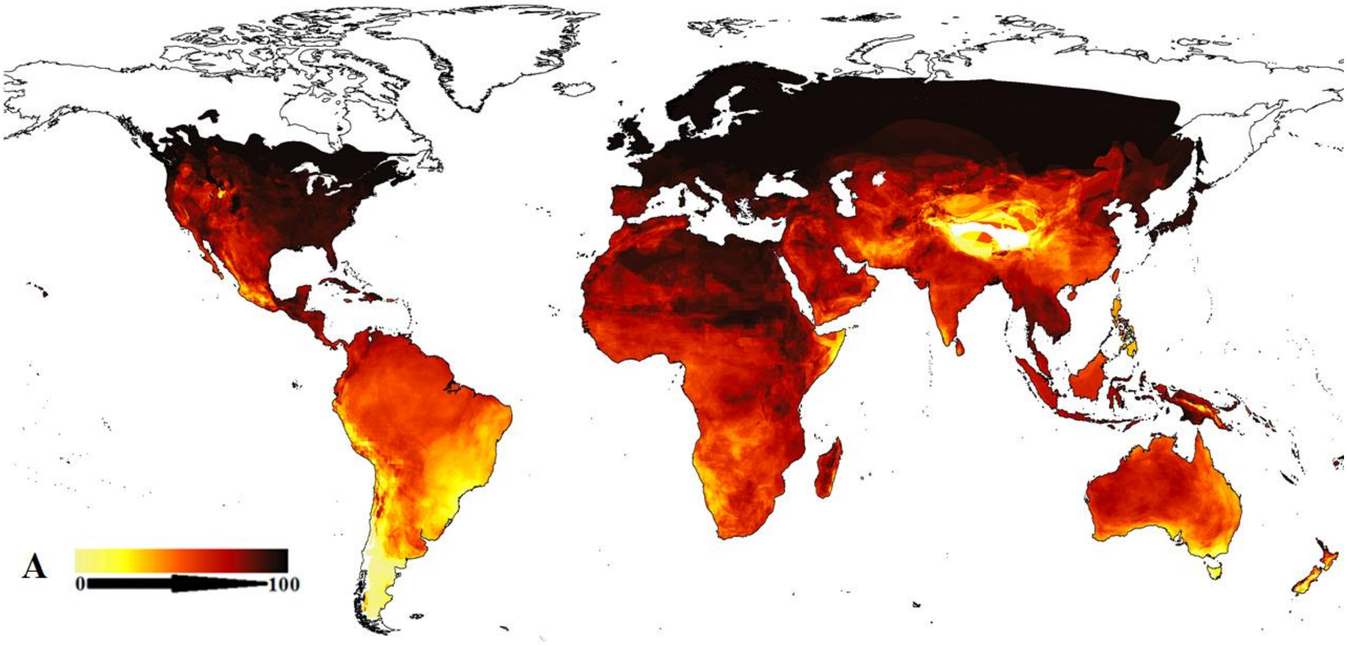
A
0
100

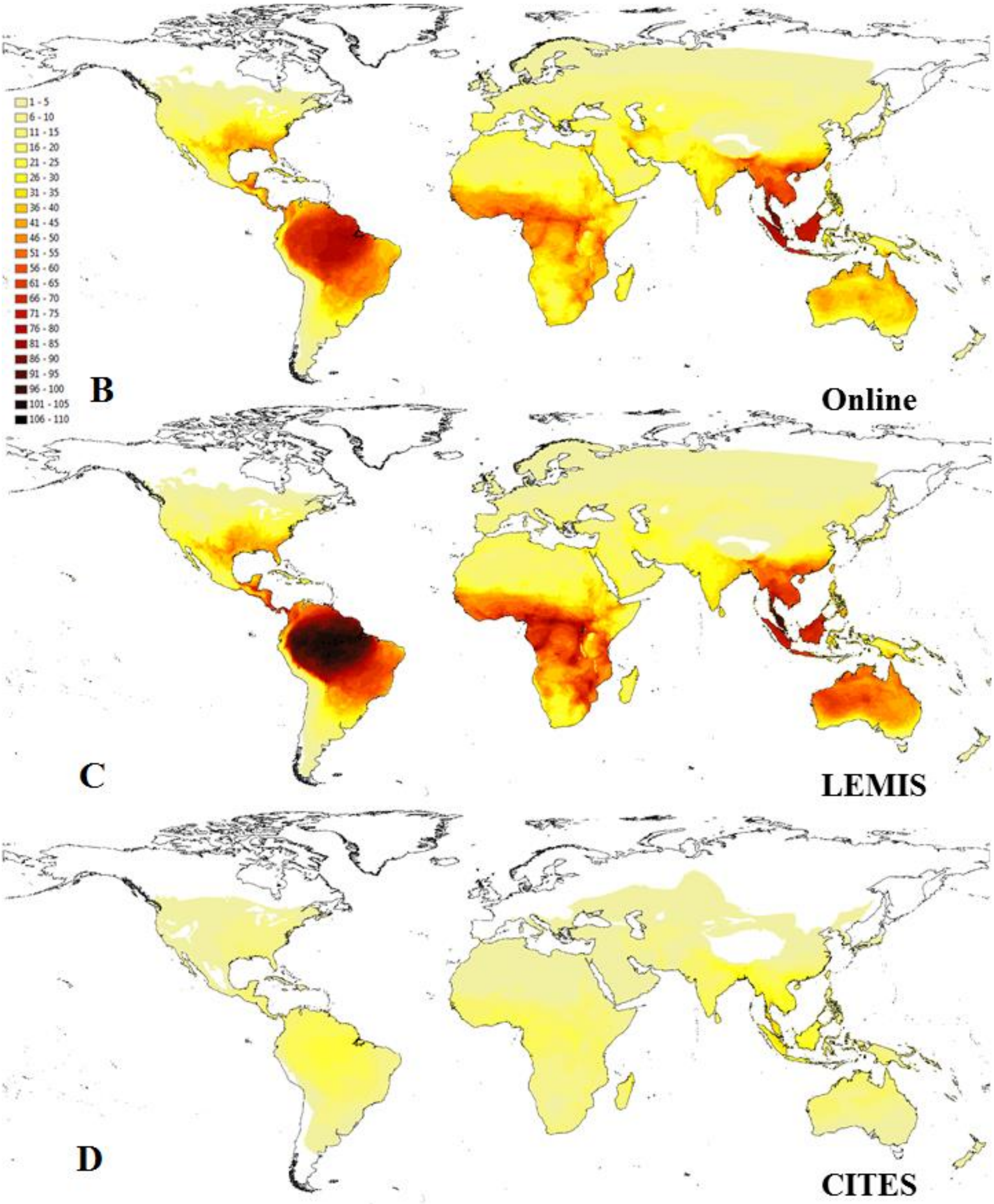

Supplementary Figure 5. A) Percentage of reptile species in trade for all areas. **B-D)** The Number of species in trade in each of the three sources, the same legend is presented in each to enable direct comparison

between the number of species exploited from each source. CITES shows notably fewer species in trade (maximum of 40) from any given area than either of the other databases (Online 98, LEMIS 108).

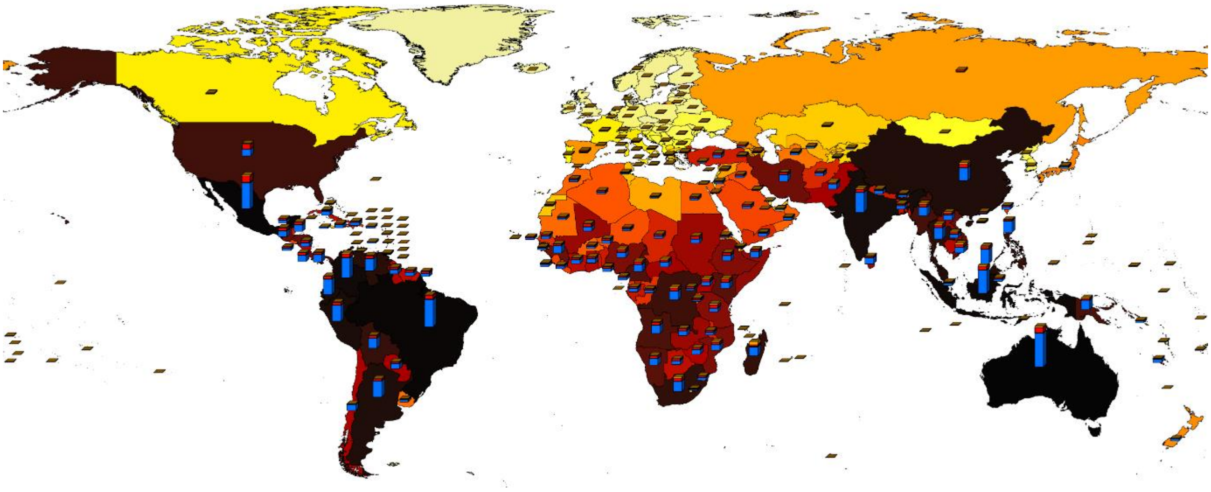

A.

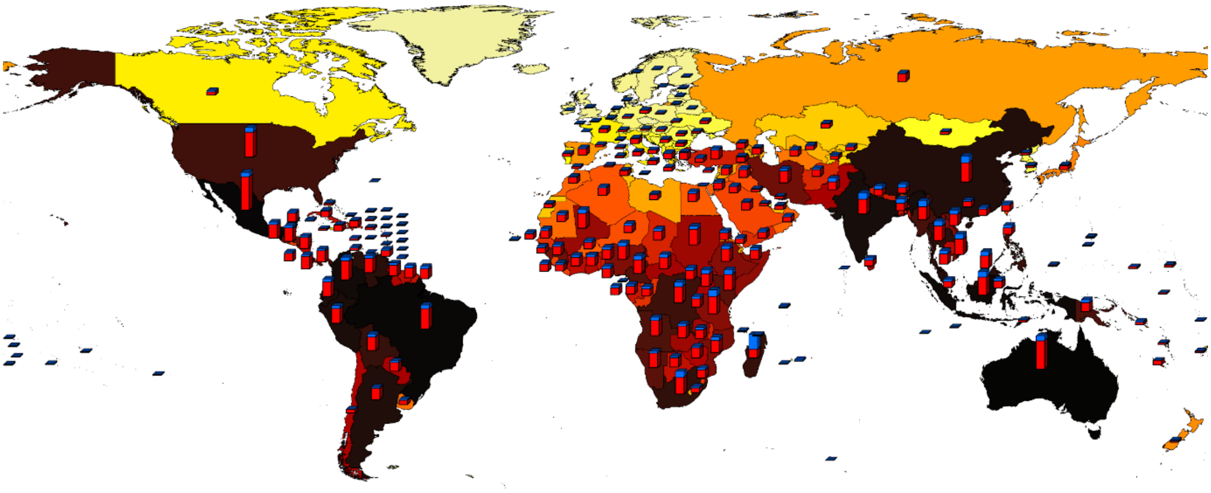

B.

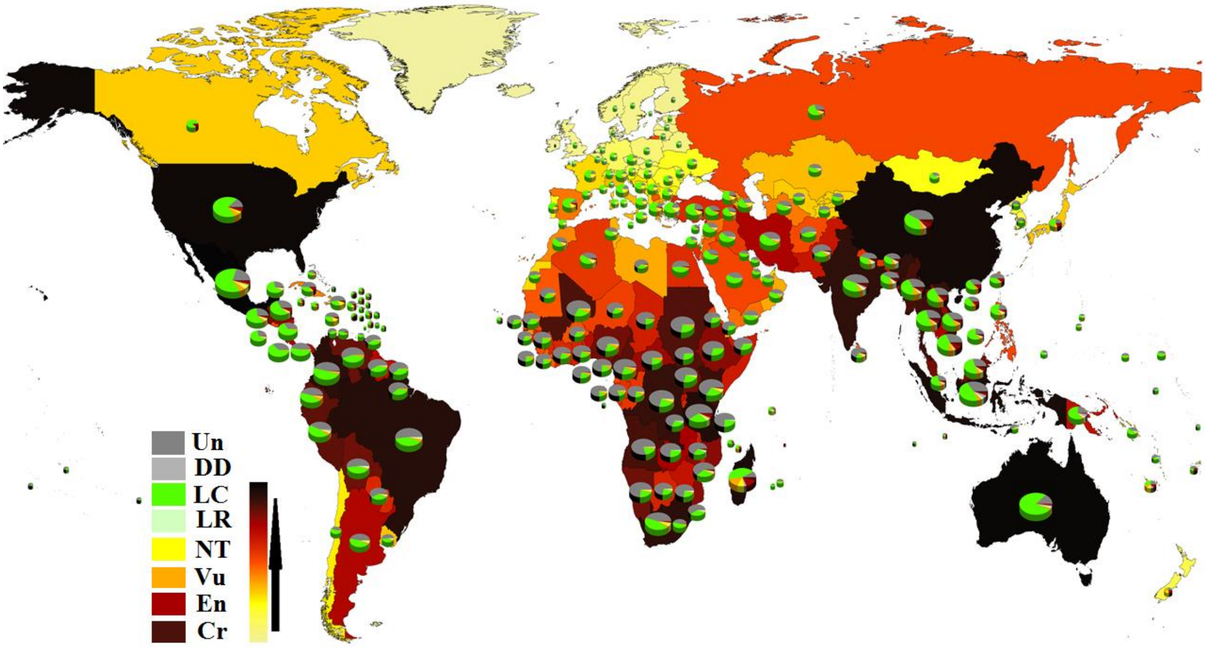

C.

Supplementary Figure 6. Number of species per administrative area and the number showing each trade status or IUCN RedList category represented as a bar or pie chart. A) Status of all species is shown on the bar (Blue: Untraded no appendix, Red-traded, no CITES-appendix, Yellow-traded, CITES-appendix, Green-untraded, appendix. **B)** Bars only show species in trade. Blue-traded with a CITES-appendix, Red-traded with no CITES-appendix. **C)** Number of species traded with each IUCN status per administrative area, the total number of species traded is shown by the size of each pie chart (Data S6).

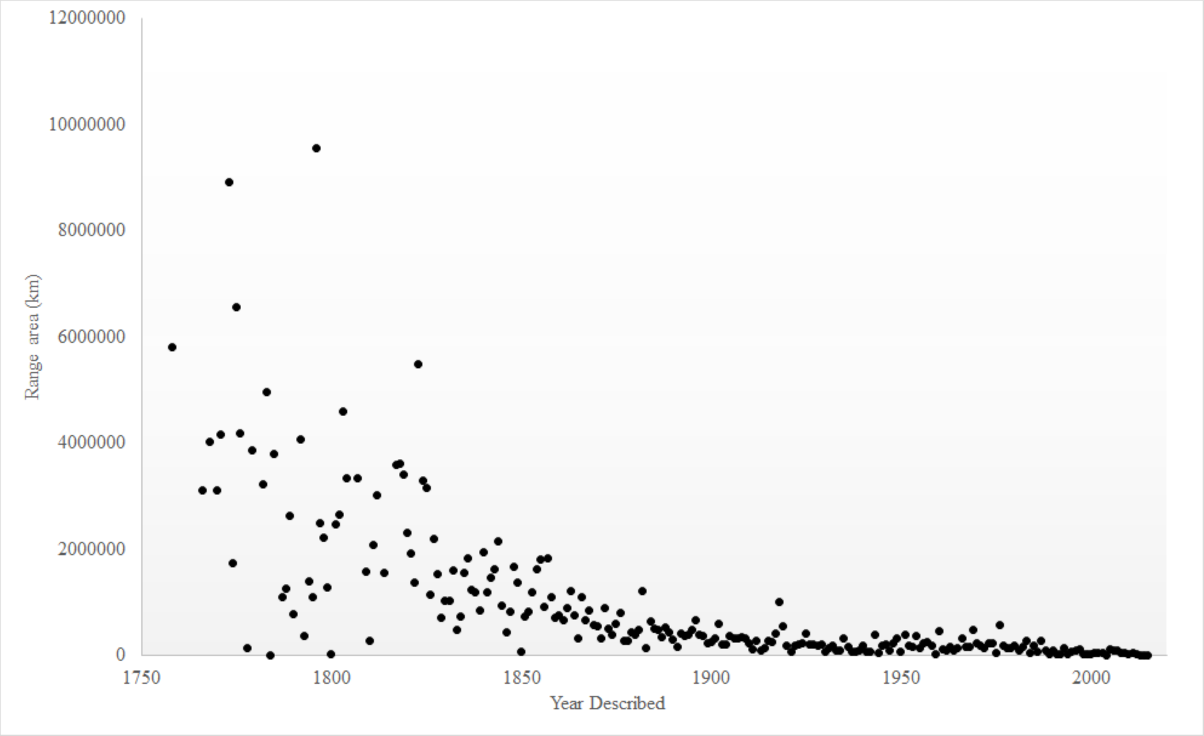

Supplementary Figure 7. The relationship between year described and the range of the described species.

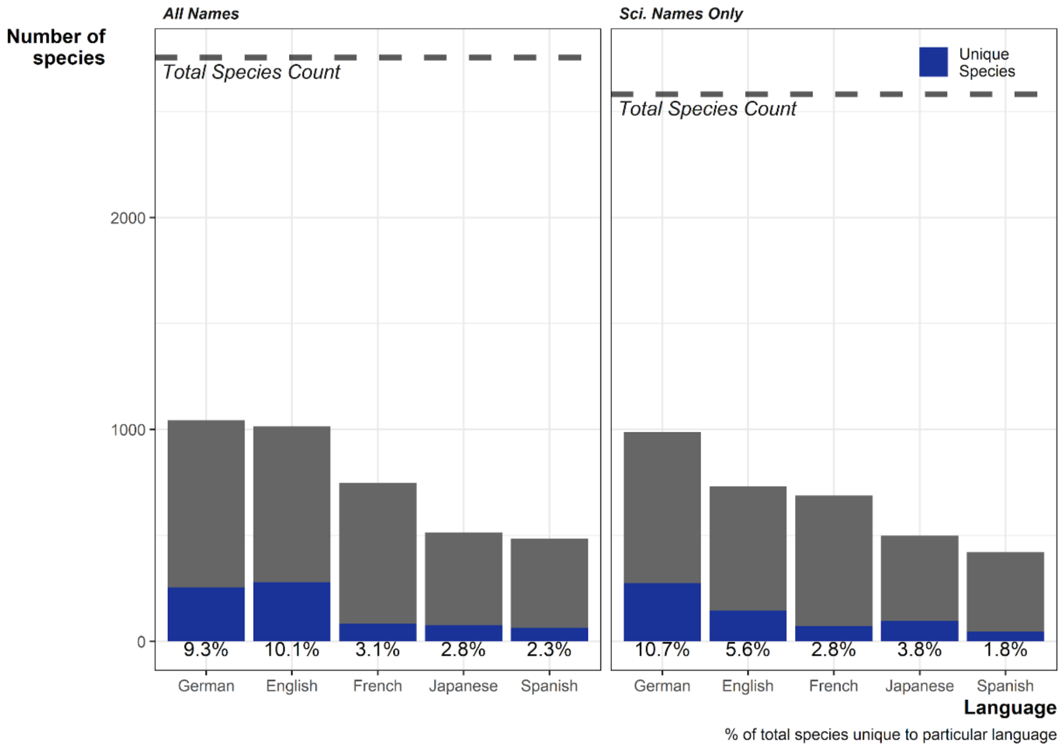

Supplementary Figure 8. The number of species detected in the online trade (2019 snapshot) split between the five languages surveyed. Blue highlights show the percentage of species unique to each language.

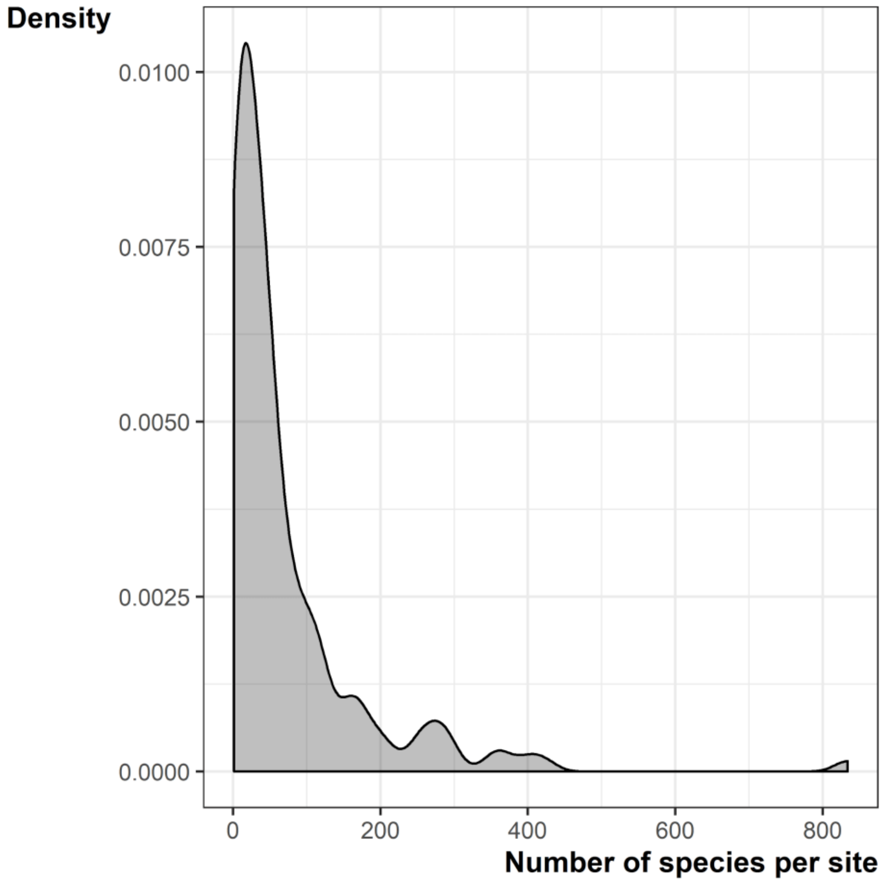

Supplementary Figure 9. Distribution of the number of species detected on a website.

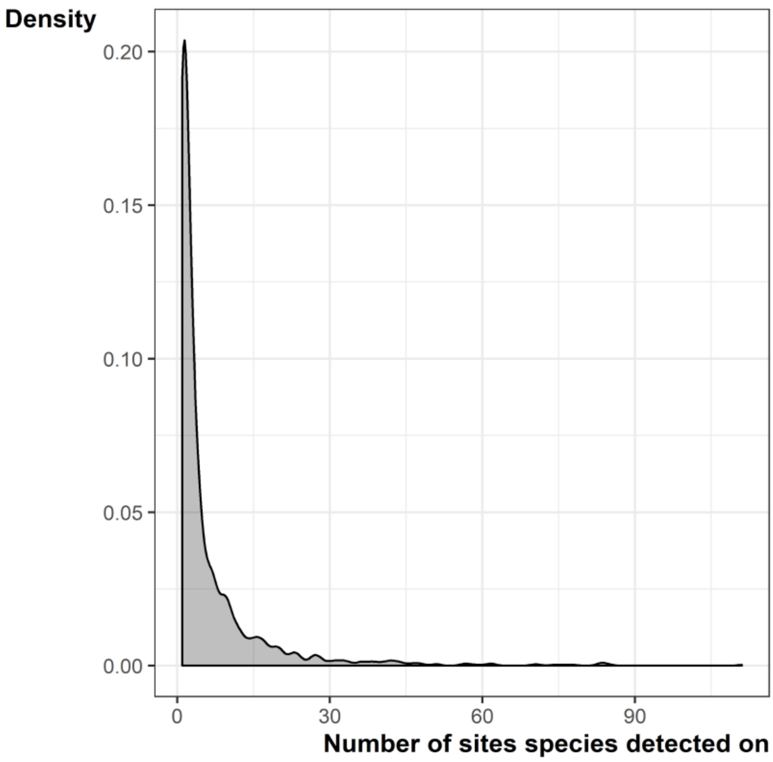

Supplementary Figure 10. Distribution of the number of websites a species was detected on.

Supplementary References

1 J. R. Harrison, D. L. Roberts, J. Hernandez-Castro, Assessing the extent and nature of wildlife trade on the dark web: Wildlife Trade on the Dark Web. Conservation Biology. 30, 900–904 (2016).

2 D. L. Roberts, J. Hernandez-Castro, Bycatch and illegal wildlife trade on the dark web. Oryx. 51, 393–394 (2017).

3 International Fund for Animal Welfare (IFAW), "Disrupt wildlife cybercrime: Uncovering the scale of online wildlife trade", 1–51, (2018).

4 E. Di Minin, C. Fink, H. Tenkanen, T. Hiippala, Machine learning for tracking illegal wildlife trade on social media. Nature Ecology and Evolution. 2, 406–407 (2018).

5 E. Di Minin, C. Fink, T. Hiippala, H. Tenkanen, A framework for investigating illegal wildlife trade on social media with machine learning. Conservation Biology. 33, 210–213 (2019).

6 J. Hernandez-Castro, D. L. Roberts, Automatic detection of potentially illegal online sales of elephant ivory via data mining. PeerJ Computer Science. 1, e10 (2015).

7 Q. Xu, J. Li, M. Cai, T. K. Mackey, Use of Machine Learning to Detect Wildlife Product Promotion and Sales on Twitter. Front. Big Data. 2, 28 (2019).

8 S. Alfino, D. L. Roberts, Code word usage in the online ivory trade across four European Union member states. Oryx, 1–5 (2018).

9 Y. Xiao, J. Guan, X. Ling, "Wildlife cybercrime in China: E-commerce and social media monitoring in 2016," TRAFFIC Briefing (TRAFFIC, 2017), (available at www.traffic.org).

10 F. M. Ratsoavina, A. P. Raselimanana, M. D. Scherz, A. Rakotoarison, J. H. Razafindraibe, F. Glaw, M. Vences, Finaritra! A splendid new leaf-tailed gecko (Uroplatus) species from Marojejy National Park in north-eastern Madagascar. Zootaxa. 4545, 563 (2019).
