# Supplementary material for: Thousands of reptile species threatened by under-regulated global trade
Source: Nat Commun. 2020 Sep 29;11:4738. doi: 10.1038/s41467-020-18523-4 (PMC7525537; doi:10.1038/s41467-020-18523-4)
Supplement: Supplementary file 3 — Reporting Summary [file 41467_2020_18523_MOESM3_ESM.pdf]

nature research

Corresponding author(s): Double-blind peer review submissions: write DBPR and your manuscript number here instead of author names.

Last updated by author(s): Jul 5, 2020

Reporting Summary

Nature Research wishes to improve the reproducibility of the work that we publish. This form provides structure for consistency and transparency in reporting. For further information on Nature Research policies, seeAuthors & Referees and theEditorial Policy Checklist .

Statistics

For all statistical analyses, confirm that the following items are present in the figure legend, table legend, main text, or Methods section.

n/a	Confirmed	
☐	☒	The exact sample size (*n*) for each experimental group/condition, given as a discrete number and unit of measurement
☐	☒	A statement on whether measurements were taken from distinct samples or whether the same sample was measured repeatedly
☒	☐	The statistical test(s) used AND whether they are one- or two-sided<br>*Only common tests should be described solely by name; describe more complex techniques in the Methods section.*
☒	☐	A description of all covariates tested
☒	☐	A description of any assumptions or corrections, such as tests of normality and adjustment for multiple comparisons
☐	☒	A full description of the statistical parameters including central tendency (e.g. means) or other basic estimates (e.g. regression coefficient) AND variation (e.g. standard deviation) or associated estimates of uncertainty (e.g. confidence intervals)
☒	☐	For null hypothesis testing, the test statistic (e.g. *F*, *t*, *r*) with confidence intervals, effect sizes, degrees of freedom and *P* value noted<br>*Give P values as exact values whenever suitable.*
☒	☐	For Bayesian analysis, information on the choice of priors and Markov chain Monte Carlo settings
☒	☐	For hierarchical and complex designs, identification of the appropriate level for tests and full reporting of outcomes
☒	☐	Estimates of effect sizes (e.g. Cohen's *d*, Pearson's *r*), indicating how they were calculated

Our web collection on statistics for biologists contains articles on many of the points above.

Software and code

Policy information about availability of computer code

Data collection	We used Firefox (70.0) to perform Google and Bing web searches for reptile selling websites. We used R (3.5.3) and R studio (1.2.1335), with a range of packages to retrieve keyword hits. The packages we used are as following: dplyr (0.8.4), stringr (1.4.0), forcats (0.4.0), tidyr (1.0.2), XML (3.99.0.3), xml2 (1.2.2), rvest (0.3.5), Rcrawler (0.1.9.1), downloader (0.4), assertthat (0.2.1), lemis (1.1.0), wayback (0.4.0), lubridate (1.7.4), jsonlite (1.6.1), and tibble (2.1.3). The code we used to generate the datasets examined is available in the supplementary material. Website names/URLs have been redacted to preserve their anonymity.
Data analysis	We used R (3.5.3) and R studio (1.2.1335), with a range of packages to examine, organise, and analyses the data. The packages we used are as following: dplyr (0.8.4), stringr (1.4.0), similiars (0.1.0), forcats (0.4.0), tidyr (1.0.2), ggrepel (0.8.1), lubridate (1.7.4), jsonlite (1.6.1), tibble (2.1.3), pracma (2.2.5), iNEXT (2.0.19), ggpubr (0.2), ggforce (0.3.1), ggplot2 (3.2.1), and scico (1.1.0). The code used to undertake analysis and generate figures is available in the supplementary material. Website names/URLs have been redacted to preserve their anonymity. We used ArcMap (10.3), ArcCatalog, and QGIS for mapping and spatial summaries.

For manuscripts utilizing custom algorithms or software that are central to the research but not yet described in published literature, software must be made available to editors/reviewers. We strongly encourage code deposition in a community repository (e.g. GitHub). See the Nature Research guidelines for submitting code & software for further information.

Data

Policy information about availability of data

All manuscripts must include a data availability statement. This statement should provide the following information, where applicable:

- Accession codes, unique identifiers, or web links for publicly available datasets
- A list of figures that have associated raw data
- A description of any restrictions on data availability

Original data generated from our online trade survey (Data S1-S4), alongside a compiled datasheet of traded species (online trade, CITES, LEMIS) is available for download as supplementary material (Data S5). Website names/URLs have been redacted to preserve their anonymity.

Field-specific reporting

Please select the one below that is the best fit for your research. If you are not sure, read the appropriate sections before making your selection.

☐ Life sciences ☐ Behavioural & social sciences ☒ Ecological, evolutionary & environmental sciences

For a reference copy of the document with all sections, see nature.com/documents/nr-reporting-summary-flat.pdf

Ecological, evolutionary & environmental sciences study design

All studies must disclose on these points even when the disclosure is negative.

Study description

This study uses three data sources to highlight the scale of the global reptile trade. The three data sources comprised of two trade databases (CITES and LEMIS) and a search of reptile selling websites. The goal was to produce a list of species being traded, and determine how many species were covered by CITES international trade regulations.

Research sample

The two trade databases (CITES and LEMIS) were chosen because they are the most comprehensive wildlife trade databases available. Both provided data freely, with minimal requirements for access. CITES trade database contains global data on CITES monitored species, mainly legal regulated trade but includes some seizures. LEMIS trade database details the animal imports into the USA, these records are known declared trade events, with a small minority detailing seized imports.

The online trade search used the search results from two search engines, Google and Bing, to generate a sample of potential reptile selling websites in 2019. We completed searches in five languages (English, French, German, Japanese, Spanish) on appropriately localised versions of the search engines. From the 679 retrieved websites, B.M.M. reviewed each one of them to confirm they sold reptiles, check whether the website terms and conditions did not explicitly forbid automated data collection, and identify the most appropriate method of searching the content of the website. 151 met the criteria and were included in our sample.

In addition to the contemporary search of reptile websites, we searched for archived pages of our most species-rich website in the 2019 sample using the Internet Archive's Wayback machine API. Here we searched every available page that pertained to the sale of reptiles. The 2019 and archived samples provided two dimensions of the online reptile trade, and covered the largest markets; therefore adequately representing the global market.

Sampling strategy

No prior sample size calculations were performed. We based our sample on what we felt would be sufficient to characterise the reptile trade, while keeping the time to review and keyword search practical.

To strengthen the representativeness of our sample, we used multiple search engines to determine the initial sample. The overlap between the results indicated we retrieved a many of the prominent online reptile sellers. Our use of five languages meant we collected data from websites originating in N. America, S. America, Eurasian and Australasia. Therefore, the sample covers the majority of market centres for the reptile trade. Practical limitations meant we examined websites from 10 pages per search engine. We believe our sample is sufficient because of the number of irrelevant websites rejected (ie we covered the major sellers), the overlap between search results (i.e. zero overlap would have resulted in >1000 websites not 679), and the similarities between online trade results and LEMIS trade data.

Data collection

After retrieving our website sample, 151 sites (23,970 pages) + archived pages (4,668), we used the stringr package to detect fixed keyword matches (code written by B.M.M.). A keyword list was compiled from all scientific and common names listed on Reptile Database and in the CITES appendices. We recorded keyword detections alongside page number, websites, the official species names, and the date connected to the page. We saved data from this procedure as csv or Rds files as described in the code supplements.

LEMIS data was retrieved by B.M.M. using the lemis package in R.

CITES trade database data was retrieved from https://trade.cites.org/# by A.C.H. IUCN Red List data was downloaded from https://www.iucnredlist.org/search by A.C.H. Global country data was downloaded from http://thematicmapping.org/downloads/world_borders.php by A.C.H.

Timing and spatial scale

We undertook the 2019 website review and sampling between 2019-09-19 and 2019-10-31. We searched for archived web page between 2019-11-14 to 2019-11-15. The resulting sample covered web pages from 2002 to 2019. Online trade data represents findings from five languages, and websites from across the globe.

LEMIS data covered a period from 2000 to 2014 and represents trade into the USA. CITES trade data spanned 2004 to 2019 and represents global CITES regulated trade. More CITES data was available, but we only examined trends that were contemporary with our other data sources.

Data exclusions

We excluded data archived webpage results linked to years 2002, 2003 and 2019 from the linear regression because they had considerably fewer pages than all other years (mean of 3.7 ±1.2 pages, compared to mean of 296.6 ±48.8 pages). This was not established exclusion criteria. All overall counts used all online data. We excluded species from LEMIS and CITES sources if they could not be confidently connected to a Reptile Database name.

Reproducibility

Reproducibility was not tested, but ad hoc manual checks of websites were undertaken to verify that keyword searches were actually detecting species.

Randomization

No randomization was required for this study, as we never assigned samples to groups.

Blinding

Blinding was not required for this study because the analysis was exploratory and largely descriptive. Decisions regarding data generation, summary, and display are visible in supplied code.

Did the study involve field work? ☐ Yes ☒ No

Reporting for specific materials, systems and methods

We require information from authors about some types of materials, experimental systems and methods used in many studies. Here, indicate whether each material, system or method listed is relevant to your study. If you are not sure if a list item applies to your research, read the appropriate section before selecting a response.

Materials & experimental systems

n/a	Involved in the study
☒	☐ Antibodies
☒	☐ Eukaryotic cell lines
☒	☐ Palaeontology
☒	☐ Animals and other organisms
☒	☐ Human research participants
☒	☐ Clinical data

Methods

n/a	Involved in the study
☒	☐ ChIP-seq
☒	☐ Flow cytometry
☒	☐ MRI-based neuroimaging
