# Supplementary material for: Thousands of reptile species threatened by under-regulated global trade
Source: Nat Commun. 2020 Sep 29;11:4738. doi: 10.1038/s41467-020-18523-4 (PMC7525537; doi:10.1038/s41467-020-18523-4)
Supplement: Supplementary file 4 — Description of Additional Supplementary Files [file 41467_2020_18523_MOESM4_ESM.pdf]

Description of Additional Supplementary Files

File Name: Supplementary Data 1
Description: Example of datasheet fed to the web searching code.

File Name: Supplementary Data 2
Description: Full list of species keywords used in the online trade search, where each row equals a separate species.

File Name: Supplementary Data 3
Description: Raw data results from the 2019 sample of online reptile selling websites. sp = Reptile Database species name; page = number of page keyword was detected on, n = number of times the keyword is detected on the page, site = website name but replaced with anonymous index, timestamp.parse = placeholder 2019 date for snapshot sample, dataset = field indicating whether data was from the 2019 snapshot or the archived web page search.

File Name: Supplementary Data 4
Description: Raw data results from the temporal sample of online the most species-rich reptile selling website. sp = Reptile Database species name; page = number of page keyword was detected on, n = number of times the keyword is detected on the page, site = website name but replaced with anonymous index, timestamp.parse = date that was supplied alongside archived web page, dataset = field indicating whether data was from the 2019 snapshot or the archived web page search.

File Name: Supplementary Data 5
Description: Resulting table detailing the full list of traded species (Reptile Database name standard), metadata concerning the species from the IUCN, their IUCN RedList status, the source identifying them as traded (online search, LEMIS, CITES), their CITES appendix (multiple entries indicating multiple appendix matches), the estimated range of the species (km2), and the year they were described. 0 = no match or not detected in a source.

File Name: Supplementary Data 6
Description: Table showing percentage of species listed in each appendix and unlisted in trade from each country, in addition to the IUCN RedList status of species being traded as a proportion of all species and as a proportion of those being traded.

File Name: Supplementary Data 7
Description: CITES and LEMIS data
Reptile_checklist_2019_08.csv – Data used in this analysis filtered to provide all fields relevant to analysis. The species list downloaded from Reptile Database that formed the taxonomic backbone for the study.

Index_of_CITES_Species_2019-09-06 04_33.csv – The list of CITES appendices and the reptile species included.

lemis_reptile_data.csv – The "Reptilia" filtered LEMIS data downloaded via the lemis R package. 14
all_traffic_04-18.csv – All reptile species list appearing in the CITES trade database

History_of_CITES_Listings_2019-12-13 04_07.csv – Data on the history CITES appendices changes. (used for temporal trend so changes in CITES listings of species could be reflected, though no species changed during the study period)

File Name: Supplementary Software 1
Description:
Supplementary Code 1. Code used to extract search results URLs;

Supplementary Code 2. Code used to compile and generate complete species search list;

Supplementary Code 3. Code used to retrieve online trade website data;

Supplementary Code 4. Code used to search websites html

Supplementary Code 5. Code used to compile and review online trade results;

Supplementary Code 6. Code used to retrieve LEMIS data;

Supplementary Code 7. Code used to retrieve archived web pages and examine the trend over time;

Supplementary Code 8. Code used to examine the lag time between species description and their appearance in the trade;

Supplementary Code 9. Code used to generate guides to Fig. 1 Venn diagram and further data review.
